# Hijacking of the Host Ubiquitin Network by *Legionella pneumophila*

**DOI:** 10.3389/fcimb.2017.00487

**Published:** 2017-12-05

**Authors:** Jiazhang Qiu, Zhao-Qing Luo

**Affiliations:** ^1^Center of Infection and Immunity, First Hospital, Jilin University, Changchun, China; ^2^Key Laboratory of Zoonosis, Ministry of Education, College of Veterinary Medicine, Jilin University, Changchun, China; ^3^Department of Biological Sciences, Purdue Institute for Inflammation, Immunology and Infectious Diseases, Purdue University, West Lafayette, IN, United States

**Keywords:** type IV secretion, effectors, posttranslational modification, bacterial virulence, cell signaling

## Abstract

Protein ubiquitination is critical for regulation of numerous eukaryotic cellular processes such as protein homeostasis, cell cycle progression, immune response, DNA repair, and vesicular trafficking. Ubiquitination often leads to the alteration of protein stability, subcellular localization, or interaction with other proteins. Given the importance of ubiquitination in the regulation of host immunity, it is not surprising that many infectious agents have evolved strategies to interfere with the ubiquitination network with sophisticated mechanisms such as functional mimicry. The facultative intracellular pathogen *Legionella pneumophila* is the causative agent of Legionnaires' disease. *L. pneumophila* is phagocytosed by macrophages and is able to replicate within a niche called Legionella-containing vacuole (LCV). The biogenesis of LCV is dependent upon the Dot/Icm type IV secretion system which delivers more than 330 effector proteins into host cytosol. The optimal intracellular replication of *L. pneumophila* requires the host ubiquitin-proteasome system. Furthermore, membranes of the bacterial phagosome are enriched with ubiquitinated proteins in a way that requires its Dot/Icm type IV secretion system, suggesting the involvement of effectors in the manipulation of the host ubiquitination machinery. Here we summarize recent advances in our understanding of mechanisms exploited by *L. pneumophila* effector proteins to hijack the host ubiquitination pathway.

## Introduction

Post-translational modification (PTM) is a biochemical mechanism in which amino-acid residues in a protein are covalently modified by specific enzymes. PTMs regulate the function of most proteins, thereby allowing the modulation of a wide range of cellular processes, which permits cells to respond to endogenous developmental signals or external stimuli imposed by environmental changes. More than 200 types of PTM have been described, including ubiquitination which is among one of best studied (Deribe et al., 2010).

### The eukaryotic ubiquitination network

**Figure 1:**
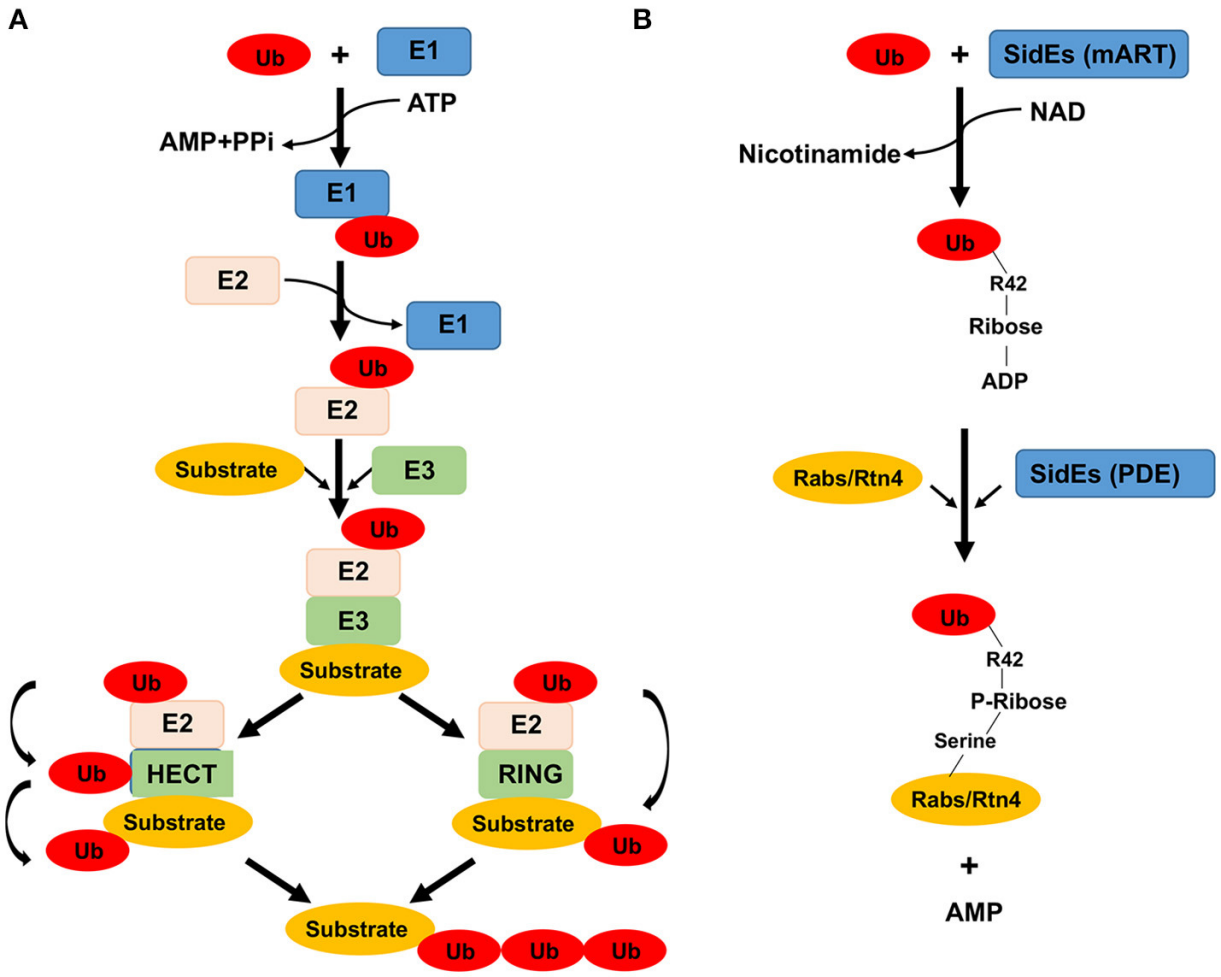
Enzymes and chemical reactions involved in ubiquitination catalyzed by the canonical mechanism and by members of the SidE family proteins. **(A)** In the canonical mechanism, a ubiquitin molecule is activated by E1 at the expense of an ATP. The activated ubiquitin is first linked to E1 via a labile thioester bond prior to being transferred to the E2 conjugation enzyme, also linked by a thioester bond. The final step of the reaction differs greatly among different groups of E3 enzymes which dictate substrate specificity. For members of the HECT family of E3 enzymes (left), a reaction intermediate is formed, again by the formation of a thiolester bond between ubiquitin and E3, from where it is finally linked to lysine residues of the substrate. For other groups of E3 enzymes such as the RING family, the ubiquitin moiety is directly transferred to the substrate without the formation of an intermediate. **(B)** The reaction catalyzed by the SidE family begins with ubiquitin activation by ADP-ribosylation at Arg_42_ to produce the reaction intermediate ADP-ribosylated ubiquitin (ADPR-Ub), a nicotinamide moiety is released in this step of the reaction. In the second reaction, ADPR-Ub is cleaved by a phosphodiesterase (PDE) activity also embedded in these proteins, resulting in the attachment of phosphoribosylated ubiquitin to serine residues of the substrate and the release of AMP. How ubiquitin is recognized by the mART motif is unknown, nor is the mechanism of substrate recognition presumably by the PDE domain.

Ubiquitination is a central signaling system that is conserved among all eukaryotic organisms (Hershko and Ciechanover, 1998; Figure 1A). Ubiquitination is defined as the covalent conjugation of one or several ubiquitin moieties to residues (mostly lysines) of target proteins. The conventional conjugation of proteins with ubiquitin occurs through the universally conserved three-enzyme cascade (Hershko and Ciechanover, 1998). Free ubiquitin is first activated by E1 (ubiquitin-activating enzyme) at the expense of ATP to form a ubiquitin-AMP intermediate that is used to modify E1 by a thiol-ester linkage formed between the carboxyl-terminus of ubiquitin and a cysteine residue on E1. The E1-linked ubiquitin is then transferred via a transthiolation reaction to a cysteine residue on E2 (ubiquitin-conjugating enzyme). Finally, E3 (ubiquitin-protein ligase) catalyzes the covalent attachment of ubiquitin to substrate via a isopeptide bond formed between the C-terminal end of ubiquitin to the ε-amino group, mostly on a lysine residue (Hershko and Ciechanover, 1998). In eukaryotic cells, there are two genes that encode E1 enzymes and dozens of genes encode E2 enzymes (Ye and Rape, 2009). Since E3 enzymes play an important role in determining substrate specificity, there are a large number of genes (over 1,000 in human genome in estimation) encoding E3 enzymes (Rytkönen and Holden, 2007). The large number of E3 ligases are classified into three major types according to the presence of different catalytic motifs and the mechanisms of catalysis. Members of the HECT (Homologous to the E6AP C-terminus) domain family E3 ligases require the formation of a thiol-ester intermediate with ubiquitin on the active cysteine residue prior to being transferred to substrates (Metzger et al., 2012). Members of the RING (really interesting new *g*ene) family E3 ligases function as adaptors that bind to both E2 and the substrate, thereby facilitating the direct transfer of ubiquitin molecule from E2 to the substrate (Metzger et al., 2012). The RING–IBR (In-Between-RINGs)–RING (RBR) type of E3s catalyze ubiquitination through a RING-HECT hybrid mechanism (Wenzel et al., 2011; Metzger et al., 2014).

The effect of ubiquitination to a large extent depends on the length and linkage type of the ubiquitin chain attached to the protein. Based on the length of the ubiquitin chain, ubiquitination can be divided into mono-ubiquitination, multi-monoubiquitination, and polyubiquitination. Monoubiquitination and multi-monoubiquitination have been shown to regulate subcellular protein localization, endocytosis, and the recruitment of ubiquitin-binding proteins (Haglund and Dikic, 2005). The formation of polyubiquitin chains can occur on one of the seven lysine residues (K6, K11, K27, K29, K33, K48, and K63) and the amino terminal methionine (M1) (Komander and Rape, 2012). Polyubiquitin chains linked via K48 render the modified proteins to be recognized by the proteasome for destruction. In contrast, polyubiquitin chains conjugated via K63 controls a wide range of important cellular signaling involved in processes such as DNA repair, endocytosis, vesicle trafficking, immunity, and cell cycle progression (Haglund and Dikic, 2005).

Ubiquitination is a reversible process catalyzed by a group of proteins known as deubiquitinating enzymes or deubiquitinases (DUBs) that cleave the isopeptide bond between ubiquitin and the modified protein. Therefore, DUBs act to recycle ubiquitin and restore the ubiquitinated substrate back to its original form. The human genome is predicted to encode nearly 100 DUBs, which according to the mechanism of action, are classified into five different families: the ubiquitin-C-terminal hydrolases (UCHs), ubiquitin-specific proteases (USPs), Machado-Joseph domain (MJD) DUBs, ovarian-tumor (OTU) domain DUBs, and the Jab1/Mov34/Mpr1 Pad1 N-terminal+ (MPN+) (JAMM) domain proteases (Wilkinson, 2009).

### Subversion of ubiquitination by bacterial pathogens

Prokaryotic cells do not possess genes coding for ubiquitin, therefore the prototypical ubiquitination pathway is absent in bacteria. Proteins modified by ubiquitination are critical regulators in virtually every eukaryotic cellular process. Thus, effective hijacking of the host ubiquitination system is essential for the success of many pathogens in their evasion of host immunity or their exploitation of host resources. For symbiotic and pathogenic bacteria, such exploitation is achieved by virulence factors that are either secreted into the extracellular milieu (which enter the host cells via various mechanisms) or are directly translocated into the cytosol of host cells via specialized secretion systems (e.g., Type III and Type IV secretion systems; Ashida et al., 2014). Some examples are species of *Shigella*, enteropathogenic *Escherichia coli* (EPEC), as well as pathogenic species of *Salmonella, Legionella* and *Chlamydia*. Accumulating evidence has shown that bacterial effector proteins exploit the host ubiquitination machinery by diverse strategies (Ashida et al., 2014; Zhou and Zhu, 2015).

The study of how bacteria co-opt the host ubiquitination machinery is a rapidly growing research field, and great progress has been made in past decades (Ashida et al., 2014). In this review, we will focus on discussing the current knowledge of effectors utilized by *Legionella pneumophila* to interfere with host ubiquitination signaling pathways. Strategies used by other human or plant bacterial pathogens will not be covered, and readers are referred to other excellent reviews (Rytkönen and Holden, 2007; Ashida et al., 2014; Zhou and Zhu, 2015; Lin and Machner, 2017).

### Intracellular replication of *Legionella pneumophila*

*L. pneumophila* is an opportunistic human pathogen that causes Legionnaires' disease, a form of potentially fatal pneumonia (Rowbotham, 1980). The genus *Legionella* was originally described in 1979 after the bacterium was identified following an outbreak of lethal pneumonia that affected participants of the 1976 American Legion Convention in Philadelphia (Fraser et al., 1977). *Legionella* spp. are ubiquitous environmental bacteria, found in freshwater niches and soil where they exist as parasites of unicellular eukaryotes such as amoebae, which are considered their natural hosts and the major source of evolutionary pressure (Moliner et al., 2010). Inhalation of aerosols contaminated by *Legionella* spp. by susceptible individuals can lead to lung infection due to robust intracellular replication in alveolar macrophages (Newton et al., 2010). The majority of human infections are caused by serogroup 1 of *L. pneumophila* and *L. longbeachae* (Newton et al., 2010). We will focus our discussion on *L. pneumophila*, the best-studied species of this pathogen.

The intracellular life cycle of *L. pneumophila* in human cells is similar to that in amoebae, which is characterized by quick establishment and maturation of the *Legionella*-containing vacuole (LCV) into a compartment with features typical for the rough endoplasmic reticulum (Swanson and Isberg, 1995). The maturation of this compartment is accompanied by sequential intimate interactions with organelles such as the ER, mitochondria, and ribosomes. The early LCV undergoes phosphoinositide conversion from PI(3)P to PI(4)P (Weber et al., 2014), the acquisition of ER resident proteins (Swanson and Isberg, 1995; Lu and Clarke, 2005) and expansion due to ER remodeling probably in part driven by the large GTPase Atlastin (Steiner et al., 2017). It is believed that this conversion allows the LCV to evade fusion with the lysosomal network (Isberg et al., 2009).

Intracellular replication of *L. pneumophila* depends completely on the Dot/Icm type IV secretion system (T4SS), which translocates more than 330 protein substrates into host cells (Finsel and Hilbi, 2015; Ensminger, 2016). These effectors comprise more than 10% of the genes predicted to code for proteins, which represent the largest arsenal of effectors among characterized bacterial pathogens. Considerable progress has been made in biochemical and cell biological studies of these effectors in the past decade, which revealed the manipulation of diverse host processes by sophisticated and novel mechanisms (Qiu and Luo, 2017).

The importance of the ubiquitin network in *L. pneumophila* virulence was first observed in a study aiming at identifying host factors important for its LCV formation and intracellular replication (Dorer et al., 2006). One of the targets found was Cdc48/p97 (Dorer et al., 2006), an AAA-ATPase that is critical for many ubiquitin-dependent processes including ER-associated degradation (ERAD) (Jarosch et al., 2002). Cdc48/p97 also recognizes ubiquitinated proteins, and often acts as a chaperone to facilitate the delivery of ubiquitinated proteins to the proteasome (Gallagher et al., 2014). This study also found that the LCV is decorated with ubiquitinated proteins shortly after its formation and such decoration requires the Dot/Icm transporter (Dorer et al., 2006), which suggests the co-option of host ubiquitination by Dot/Icm effectors. Here we will highlight the subversion of the host ubiquitination machinery by Dot/Icm effectors that function by mimicking known mechanisms or by unprecedented modes of action.

## *L. pneumophila* effectors that function as E3 ubiquitin ligases

Pathogen-mediated ubiquitination is mostly catalyzed by virulence factors that mimic the function of E3 ligases (Maculins et al., 2016). In the case of *L. pneumophila*, a large cohort of effector proteins are known to be involved in ubiquitination, either by mimicking classic E3 ligase families or by completely novel mechanisms (Table 1).

**Table 1:** *L. pneumophila* Dot/Icm effectors involved in ubiquitination.

**Effectors (gene number)**	**Aliases**	**Interactor/Substrate**	**Enzymatic activity**	**Function**	**References**
lpg0171	legU1	SKP1, Cullin 1, BAT3	F-Box protein, E3 ubiquitin ligase	Unknown	Ensminger and Isberg, 2010
lpg1408	licA	SKP1	F-Box protein	Unknown	Ensminger and Isberg, 2010
lpg2144/lpp2082	legAU13/ankB	SKP1, Cullin 1, Parvin B	F-Box protein, E3 ubiquitin ligase	Recruitment of polyubiquitinated species to LCV; Generation of amino acids for *L. pneumophila* replication	Price et al., 2009, 2011; Ensminger and Isberg, 2010; Lomma et al., 2010
lpg2224	PpgA	Unknown	F-Box protein	Unknown	Ensminger and Isberg, 2010
lpg2525	–	Unknown	F-Box protein	Unknown	Ensminger and Isberg, 2010
lpp2486	–	Unknown	F-Box protein	Unknown	
lpg2455	GobX	Unknown	U-Box protein, E3 ubiquitin ligase	Unknown	Lin et al., 2015
lpg2830	LegU2/LubX	Clk1, SidH	U-Box protein, E3 ubiquitin ligase	SidH degradation	Kubori et al., 2008, 2010
lpg2510 lpg2511	SdcA and SidC	Unknown	E3 ubiquitin ligase	Recruitment of ER vesicles and polyubiquitinated species to LCV	Hsu et al., 2014
lpg0234	SidE	Rab1, Rab6a, Rab30, Rab33b, Rtn4	All-in-one ubiquitin conjugation enzyme; Deubiquitinase	Intracellular replication; regulation of ubiquitin dynamics on the LCV; Recruitment of ER markers to the LCV; ER tubule Rearrangement.	Sheedlo et al., 2015; Bhogaraju et al., 2016; Qiu et al., 2016; Kotewicz et al., 2017
lpg2153	SdeC				
lpg2156	SdeB				
lpg2157	SdeA				
lpg1148	LupA	Unknown	Deubiquitinase	Unknown	Urbanus et al., 2016
lpg2155	SidJ	Rab1, Rab6a, Rab30, Rab33b, Rtn4	Phosphodiesterase, Deubiquitinase	Recruitment of ER markers to the LCV; Regulation of SidEs-mediated substrates modification	Liu and Luo, 2007; Qiu et al., 2017

### U-box and F-box E3 ligases

The RING type E3 ligases which contain a conserved RING domain constitute the large majority of known E3s in eukaryotic cells (Metzger et al., 2014). The RING domain consists of 40–60 residues and coordinates two Zn^2+^ ions in a cross-braced arrangement to form a platform for binding to E2s. RING-type domains can either exist as single-subunit proteins which tend to form homodimers and heterodimers, or as multi-subunit assemblies, including Cullin RING E3 ligase complexes (CRLs). Each CRL subfamily consists of a Cullin protein serving as scaffold, a small RING protein (in most cases Rbx1/Roc1/Hrt1), an adaptor protein and a protein for substrate binding. The best-studied CRLs are the SCF (Skp1-Cul1-F-box protein) family, which contains the RING-domain protein Rbx1, Cullin 1, SKP1 (S-phase-kinase associated protein 1), and an F-box domain-containing protein that directly binds SKP1 (Schulman et al., 2000). In addition, F-box-containing proteins are capable of recognizing specific substrates via leucine-rich repeat (LRR) or WD40 protein-binding domains. The U box is a motif of 70 amino acids that is present in proteins from yeast to humans; it is capable of assembling poly-ubiquitin chains (Hatakeyama et al., 2001). Due to the structural similarity between U-box and RING domain, U-box-containing E3 ligases are classified as RING-type E3s. The U-box E3s use intramolecular interactions other than zinc chelation to maintain the RING finger motif due to the absence of canonical cysteine residues for Zn^2+^ coordination (Hatakeyama et al., 2001). A wide range of host signaling pathways are controlled by ubiquitination catalyzed by the RING-type family E3 ligase, and this mechanism is often targeted by bacterial pathogens for their own advantage.

A study aiming at screening for genes that encode proteins with features typical for eukaryotic proteins in the *L. pneumophila* genome identified proteins that harbor domain structures known to be involved in ubiquitin manipulation. These include two proteins that harbor an F-box domain and one gene product that harbors a U-box domain (de Felipe et al., 2005). Currently, seven F-box-containing proteins (LegU1, LicA, Lpg1975/Lpp1959, AnkB/LegAU13), PpgA/Lpg2224 Lpg2525, and Lpp2486 (Only in strain Paris) and two U-box-containing proteins (LubX/LegU2 and GobX) have been identified in *L. pneumophila* strain Philadelphia 1 (Hubber et al., 2014). Without exception, these proteins are translocated to the host cytosol via the Dot/Icm machinery during infection. Although, the exact number varies, proteins that contain these domains exist in predicted effectors among all sequenced Legionella species (Burstein et al., 2016). Four of them LegU1, AnkB, LubX, and GobX have been proven to possess E3 ligase activity through biochemical studies (Kubori et al., 2008; Ensminger and Isberg, 2010; Ensminger, 2016).

**Figure 2:**
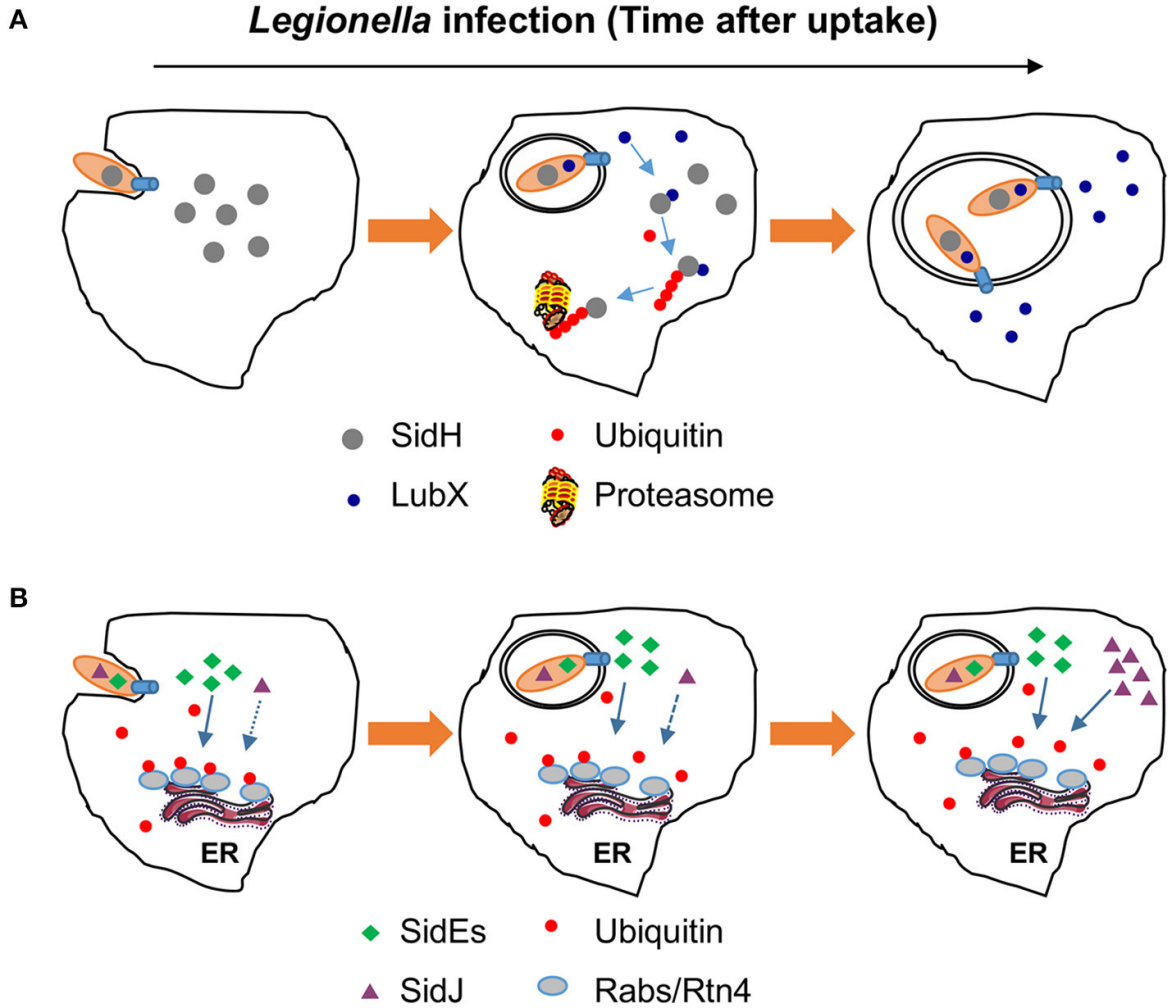
Temporal regulation of effector activity by effectors. **(A)** Regulation of SidH by LubX. The expression of *lubX* does not become apparent until after several hours postinfection. This ubiquitin E3 ligase functions with the host machinery to ubiquitinate SidH, resulting its degradation by the proteasome. **(B)** Regulation of SidEs by SidJ. SidEs catalyze the ubiquitination of RTN4 or ER-associated Rab small GTPases such as Rab33b whereas SidJ reverses such modification by its phosphodiesterase activity. In the early phase of infection, the ratio between translocated SidEs and SidJ favors ubiquitination of relevant substrates, which is beneficial for the biogenesis of the LCV. Several hours after bacterial uptake, the activity of SidJ becomes dominant due to higher amount of translocated protein, which reverses the ubiquitination imposed by the SidEs.

Effector proteins are generally thought to target host proteins. However, LubX is capable of binding and ubiquitinating SidH, another *L. pneumophila* effector protein, leading to its degradation by the proteasome (Kubori et al., 2010). LubX is thus designated as a “metaeffector,” an effector that regulates the activity of one or more other effectors. Expression and thus the translocation of LubX only occur several hours after bacterial uptake by host cells, and peaks at 10 h post infection. This delayed translocation of LubX to the host cytosol results in the shutdown of SidH within the host cells at later stages of infection, suggesting a temporal regulation of SidH activity by LubX (Kubori et al., 2010; Figure 2A). These results suggest that SidH is only beneficial for bacterial infection in the first several hours after uptake. Indeed, disruption of *lubX* led to the persistence of intracellular SidH accompanied by a hyper-lethal phenotype of *L. pneumophila* in a fly infection model (Kubori et al., 2010).

LubX contains two domains that have a remarkable similarity to the eukaryotic U-box. LubX has ubiquitin ligase activity with a preference for the UbcH5a or UbcH5c E2 enzymes (Kubori et al., 2008). Structural studies have provided more detailed insights in the molecular mechanism adopted by U-box domains of LubX (Quaile et al., 2015). The two U-box domains are structurally similar and both have adopted the typical fold of their eukaryotic counterparts. The structure of LubX in complex with E2 enzyme UBE2D2 highlighted the remarkable differences in recognizing E2 enzymes between the U-box domains within LubX. Although, the U-box folds are highly conserved, there are significant variations of residues in U-box 2 that are critical for the formation of canonical E2 binding site in most U-boxes, preclude E2 association by U-box 2 (Quaile et al., 2015). Additionally, among the surface-exposed residues in LubX, Arg_121_ was the only residue identified to be critical for the interaction between LubX and SidH (One of the *L. pneumophila* effector proteins). However, Arg_121_ localizes to the alpha C helix connecting the two U-box domains, and thus is not part of the U-box fold (Quaile et al., 2015). Notably, the U-box 2 domain employed by Kubori et al. is required for the association of SidH and also contain the alpha C helix, and thus the Arg_121_ is included (Kubori et al., 2010). Interaction of LubX and SidH might occur over a large area, and require the contribution of a number of residues for interaction. Therefore, single point mutation likely fails to disrupt the interaction surface. Instead, Arg_121_ may be important for the stabilization of the LubX structure (Quaile et al., 2015).

LubX also binds to the host factor Cdc2-like kinase 1 (Clk1) and directs its polyubiquitination *in vitro*. The N-terminal U-box domain (U-box 1) of LubX is essential for ubiquitin ligation, and serves as the E2 binding site, while the C-terminal U-box (U-box 2) is dispensable for interaction with Clk1 (Kubori et al., 2008). Thus, LubX has a non-canonical U-box domain that functions to mediate substrate recognition rather than E2 binding, which is a function not previously reported for eukaryotic U-box domains. The reason that LubX adopts a U-box domain for substrate binding is unclear. Clk kinases have been shown to interact with, and phosphorylate, serine- and arginine-rich (SR) proteins, which in turn regulate mRNA splicing (Prasad et al., 1999). Inhibition of Clk kinases interferes with intracellular growth of *L. pneumophila*, suggesting that these enzymes regulate pathways important for the development of the bacterial vacuole (Kubori et al., 2008). However, deletion of *lubX* did not cause any growth defect in mouse macrophages or in protozoan cells; in addition, over-expression of LubX in cells only ubiquitinates a small amount of Clk1 (Kubori et al., 2008). Therefore, the mechanism used by Clk1 to modulate *L. pneumophila* growth and whether Clk1 is ubiquitinated by LubX under infection conditions as well as the consequences of modification remain to be clarified.

Another Dot/Icm effector protein GobX possesses a central domain that has a secondary structure remotely similar to U-box motif. GobX exhibits E3 ubiquitin ligase activity in reactions with the E2 enzymes UbcH5a, -5b, -5c, or UbcH6 (Lin et al., 2015). GobX exhibit limited homology at the primary sequence level to other U-box domains, however, the conserved hydrophobic/aromatic residues involved in E2 interaction used by other U-box proteins are also present in the secondary structure of GobX, as mutations in Ile-58 or Trp-87 strongly attenuated its ubiquitination activity (Lin et al., 2015). In addition, the hydrophobic lipid palmitate is covalently attached to Cys_175_ of GobX, which allows the protein to specifically localize to the Golgi apparatus (Lin et al., 2015). Therefore, GobX exploits host cell S-palmitoylation to gain accurate host subcellular targeting. Similar to most Dot/Icm effector proteins, GobX is dispensable for intracellular survival and proliferation of *L. pneumophila* within host cells, which again highlights the potential functional redundancy within the effector repertoire (Lin et al., 2015). Host substrates of GobX are currently unknown, which limits our understanding of how its ubiquitination activity benefits intracellular bacterial growth.

To date, all sequenced *L. pneumophila* strains encode genes with predicted F-box domains. For example, strain Philadelphia-1 harbors five F-box motif-containing proteins (Ensminger and Isberg, 2010). The F-box is a motif that is best known for its role in interaction with other proteins such as SKP1, a core component of the SCF complex (Skaar et al., 2013). Since bacteria do not produce any SKP1, CUL1, or RBX1, *L. pneumophila* F-box proteins require SCF components provided by host cells to be functional. All the F-box motif-containing proteins are delivered into host cells during infection through the Dot/Icm apparatus (Ensminger and Isberg, 2010). Three of the *L. pneumophila* F-box proteins, LegU1, AnkB, and LicA were able to interact with SKP1 in mammalian cells, indicating the presence of a functional F-box domain within these proteins (Ensminger and Isberg, 2010). In contrast, no interaction of SKP1 was detected in cells expressing either PpgA/Lpg2224 or Lpg2525, suggesting that these proteins may not function as canonical F-box proteins (Ensminger and Isberg, 2010). Additionally, LegU1 and AnkB also interact with Cullin 1 and integrated into functional SCF complexes which may confer E3 ligase activity. Although, LicA binds to SKP1, it fails to interact with Cullin 1, suggesting that this effector is unable to form a functional E3 ligase. Alternatively, LicA may require a different set of eukaryotic proteins to form an active E3 complex (Ensminger and Isberg, 2010). In addition, due to the presence of another predicted choline kinase domain, it is also possible that LicA functions to modulate SKP1 activity (Ensminger and Isberg, 2010).

Consistent with the predicted function of F-box proteins, SCF complexes formed by LegU1 or AnkB exhibit E3 ligase activity (Ensminger and Isberg, 2010). The E2 proteins UBCH5a and UBCH5c stimulate robust formation of self-ubiquitinated LegU1 and LegAU13. LegU1 binds the host protein HLA-B-associated transcript 3 (BAT3) and specifically directs its polyubiquitination (Ensminger and Isberg, 2010). BAT3 is an abundant and highly conserved protein in higher eukaryotes; it participates in the regulation of a wide range of host processes including apoptosis, the response to ER stress, p53-regulated gene expression, and Hsp70 stability (Desmots et al., 2008; Sasaki et al., 2008). However, the biological role of LegU1-catalyzed polyubiquitination of BAT3 in *L. pneumophila* pathogenesis remains unknown. LegU1 also interacts with another effector protein Lpg2160 via the BAT3-LegU1 complex. Yet, LegU1 does not detectably ubiquitinate Lpg2160 (Ensminger and Isberg, 2010). Since both LegU1 and Lpg2160 interact with BAT3, they might have overlapping functions during *L. pneumophila* infection. The host proteins targeted by AnkB for ubiquitination are still mysterious. Lomma et al. reported that Lpp2082, the ortholog of AnkB in *Legionella* strain Paris, interacts with the host protein Parvin β/ParvB, an endogenously ubiquitinated protein (Lomma et al., 2010). Surprisingly, expression of Lpp2082 in cells led to a decrease of ubiquitinated ParvB (Lomma et al., 2010). Lpp2082 might modulate ParvB ubiquitination by competing with the interaction sites normally used by eukaryotic E3 ligases. ParvB functions as a pro-apoptotic protein (Fukuda et al., 2003; Zhang et al., 2004), and thus reduction of ParvB ubiquitination by Lpp2082 will compromise its pro-apoptotic effects. Indeed, Lpp2082 is implicated in apoptotic signaling, as supported by the evidence that infection of cells with a Lpp2082 deficient mutant strain led to a significant reduction of caspase-3 activity (Lomma et al., 2010).

It is possible that some of the F-box and U-box proteins are responsible for the enrichment of ubiquitin species on the LCV (Dorer et al., 2006). These effectors may function to facilitate the degradation of proteins by proteasome that are detrimental to intracellular bacterial growth. This notion is consistent with the fact that inhibition of proteasome activity leads to arrest in the development of the LCV (Dorer et al., 2006). In *L. pneumophila* strains AA100 and Paris, significant defects in recruitment of ubiquitinated species to LCV were observed in AA100 strain with insertion mutation of *ankB* gene or Paris strain with an in-frame deletion of *Lpp2082* (Price et al., 2009; Lomma et al., 2010). In addition, these strains also displayed striking intracellular growth defect in mouse macrophages and *Acanthamoeba castellanii* (Al-Khodor et al., 2008; Lomma et al., 2010). However, the importance of AnkB in *L. pneumophila* virulence differs greatly among different strains. In strain Philadelphia-1, single mutants lacking one of the F-box and U-box proteins which has established E3 ligase activity, including LegU1, AnkB, LegU2, did not show significant growth defect within any examined host cells (Ivanov and Roy, 2009). Strikingly, recruitment of ubiquitinated proteins to the LCV was also not affected either by single mutants or a quadruple mutant strain lacking *legU1, ankB, licA*, and *legU2* (Ivanov and Roy, 2009). The genetic background of different strains and/or subtle differences in mutant strain construction may be responsible for such discrepancy.

### New type of E3 ligases (SidC and SdcA)

The mature LCV is characterized by an enrichment of a particular phosphoinositide lipid, PI(4)P. Upon being delivered by the Dot/Icm machinery into the host cytosol, SidC is highly enriched on the LCV membrane via its PI4P binding domain located at the C-terminal end of the protein (Luo and Isberg, 2004; Ragaz et al., 2008). SidC and its paralog SdcA were shown to play a key role in recruiting ER-derived vesicles and ubiquitinated proteins onto the LCV, which requires the N-terminal domain of SidC and SdcA (Ragaz et al., 2008). Therefore, SidC and SdcA have since been considered as tethering factors for host proteins. Several groups determined the crystal structure of N-terminal domain of SidC, which showed a novel fold without resemblance to any characterized proteins (Gazdag et al., 2014; Horenkamp et al., 2014; Hsu et al., 2014). Detailed sequence homology analysis revealed a canonical Cys-His-Asp (C46, H444, and D446) catalytic triad located at the surface of SidC, a motif usually found in cysteine proteases and deubiquitinase (Hsu et al., 2014). Further study revealed that instead of acting as hydrolytic enzymes, SidC and SdcA exhibit E3 ligase activity in a mechanism that requires the C_46_-H_444_-D_446_ catalytic triad (Hsu et al., 2014). Among the several E2 enzymes tested, SidC functions most efficiently with UbcH7 and preferentially catalyzes the formation of K11 and K33-linked polyubiquitin chains (Hsu et al., 2014). SdcA shares 72% sequence identity with SidC; Yet, it prefers UbcH5 for efficient poly-ubiquitin chain assembly. The molecular mechanism of differential preference for E2 enzymes by SidC and SdcA remains to be studied. The E3 activity is essential for the recruitment of ER proteins and ubiquitinated proteins to the LCV by SidC and SdcA, as a C46A mutation abolishes this activity (Hsu et al., 2014). Further structural analysis of a larger portion of SidC that encompasses the E3 ligase domain and the PI4P binding domain (Luo et al., 2015) suggests that the PI4P binding domain masked the active site of the E3 ligase domain (Luo et al., 2015). Indeed, the activity of full-length SidC is lower than its truncation mutants lacking the PI4P-binding domain (Luo et al., 2015), suggesting that PI4P association leads to an “open” conformation where the catalytic sites of the SidC E3 ligase domain are exposed (Luo et al., 2015). Taken together, these findings suggest an intramolecular regulation model for SidC. Further, binding to PI4P may not only compartmentalize the activity of SidC and SdcA to the LCV but also maximize their activity. Such regulation would reduce non-specific protein ubiquitination and exert less unintended interference of host processes.

The observation that SidC and SdcA play important roles for the recruitment of ER-derived vesicles to the LCV suggests these E3 ligases manipulate the function of host proteins involved in vesicle trafficking. Indeed, the small GTPase Rab1, a key regulator of ER to the *cis*-Golgi trafficking, was mono-ubiquitinated during *L. pneumophila* infection in an manner that requires SidC and SdcA (Horenkamp et al., 2014). However, mono-ubiquitination of Rab1 was not detected in cells coexpressing these two proteins or in reactions containing all of the components required for the activity of SidC and SdcA (Hsu et al., 2014). Future studies need to focus on the identification of the substrates modified by SidC and SdcA, which will definitely shed light on how these E3 ligases benefit intracellular bacterial replication.

### All-in-one ubiquitin E3 ligases

The three-enzyme cascade is the fundamental principle of all described ubiquitination events, in which E1 and E2 enzymes are indispensible for the reaction to occur (Qiu et al., 2016; Figure 1A). However, recent studies of the *L. pneumophila* SidE effector family (SidEs) rewrote this strict rule of ubiquitination (Qiu et al., 2016). SidEs distinguish themselves from most of the *L. pneumophila* Dot/Icm effectors by their importance in intracellular bacterial growth in the protozoan host *Dictyostelium discoideum* (Luo and Isberg, 2004; Bardill et al., 2005). Bioinformatics analysis identified a putative mono-ADP-ribosyltransferase (mART) motif located in the middle of all SidE family proteins such as SdeA that is essential for their toxicity to yeast and for the ability to complement a mutant lacking this effector family (Qiu et al., 2016). Proteins containing an mART motif usually catalyze mono-ADP-ribosylation of arginine residues in target proteins with nicotinamide adenine dinucleotide (NAD) as the substrate (Simon et al., 2014). However, no ADP-ribosylation activity was detected in reactions containing recombinant SdeA. Surprisingly, when expressed in mammalian cells, SidEs were found to induce ubiquitination of several ER-associated Rab small GTPases including Rab33b and Rab1, in a manner that requires the mART motif (Qiu et al., 2016). Further analysis revealed that SidEs catalyze ubiquitination by a mechanism that is fundamentally different from the classical three-enzyme cascade (Qiu et al., 2016). First, the reaction is independent of the host ubiquitination machinery and does not require E1 and E2 enzymes; second, instead of ATP, it utilizes NAD as the energy source; Third, SidEs activate ubiquitin via ADP-ribosylation of Arg_42_ of the modifier molecule to produce the reaction intermediate ADP-ribosylated ubiquitin (ADPR-Ub). Consistent with this observation, the two glycine residues in the carboxyl end of ubiquitin essential for the canonical reaction are not required for the new reaction. It also suggests that the ubiquitin is linked to the substrate via a covalent bond that differs from the isopeptide bond used by most canonical reactions (Figure 1B). This discovery represents the first example of an ubiquitin-specific mART, as well as the first documentation of E1/E2 independent ubiquitination (Bhogaraju and Dikic, 2016).

Two subsequent studies revealed that ADPR-Ub produced by the mART motif is utilized by a phosphodiesterase (PDE) activity also embedded in SidEs to modify target proteins (Bhogaraju et al., 2016; Kotewicz et al., 2017). In this reaction, the phosphodiester bond between the two phosphate groups in ADPR-Ub is cleaved by a phosphodiesterase activity conferred by the PDE domain, leading to the release of AMP and attachment of phosphoribosylated ubiquitin (PR-Ub) to serine residues in the presence of target proteins or the production of the free PR-Ub when water is the acceptor molecule (Bhogaraju et al., 2016; Kotewicz et al., 2017; Figure 1B).

Ubiquitination of Rab33b by SdeA detectably affects its activity in GTP loading and hydrolysis but did not detectably affect its stability (Qiu et al., 2016). However, how ubiquitination of the Rabs by SidEs contributes to *L. pneumophila* virulence remains to be studied. Notably, SidEs appear to have multiple structurally diverse substrates in host cells. These ligases ubiquitinate reticulon 4 (Rtn4), a protein that regulates the dynamics of the tubular ER. Ubiquitination of Rtn4 causes a rearrangement in tubule ER and its enrichment on the LCV (Kotewicz et al., 2017). It is anticipated that SidEs likely attack additional host proteins. Interestingly, both ADPR-Ub and PR-Ub produced by the activity of SidEs potently impair the conventional ubiquitination reaction by blocking the activation of E1 and E2 enzymes, leading to the interference of a wide range of ubiquitination-dependent cellular events including mitophagy and TNF signaling (Bhogaraju et al., 2016). Because a SdeC mutant defective in the PDE activity was unable to restore the virulence of the *L. pneumophila* mutant lacking the SidE effector family, ubiquitination of substrates but not the interference of host normal ubiquitination events is responsible for the role of the SidEs in bacterial virulence (Kotewicz et al., 2017). Nevertheless, the strong inhibitory effects of ADPR-Ub and PR-Ub suggest that eukaryotic cells may regulate ubiquitin signaling by producing these molecules from endogenous enzymes (Bhogaraju and Dikic, 2016; Bhogaraju et al., 2016).

Although, the biochemical mechanism of SidEs-mediated ubiquitination has been largely elucidated, several questions remain. First, how do SidEs recognize ubiquitin and substrates? Second, how do the mART and PDE motifs coordinate their activity? Are these two activities channeled or do they function independently of each other? Third, how does the activity of SidEs contribute to virulence? Future structural and cell biological studies will continue to provide exciting insights into these questions.

Pathogenic bacteria, especially intracellular pathogens often acquire toxins or effector proteins by horizontal gene transfer during their coevolution with host cells. It is therefore likely that eukaryotic cells utilize mechanisms similar to that by SidEs for ubiquitination. It is possible that proteins harboring domains capable of producing and utilizing ADPR-Ub form complexes to modify their substrates. The identification of such enzymes will surely lead to better appreciation of the cellular processes regulated by ubiquitin.

## *L. pneumophila* deubiquitinases (DUBs)

Ubiquitination is a reversible process and the removal of ubiquitin from modified proteins is carried out by the action of a large family of proteases known as deubiquitinase (DUBs). DUBs specifically catalyze the cleavage of isopeptide linkage between ubiquitin and substrate or within poly-ubiquitin chains, resulting in the release of ubiquitin as well as the termination or alteration of biological events of the substrate proteins (Wilkinson, 2009). DUBs have been found to be employed by several bacterial pathogens to effectively modulate the host signaling pathway regulated by ubiquitin (Zhou and Zhu, 2015). Examples include SseL of *Salmonella enterica* Typhimurium, ChlaDub1, and ChlaDub2 by *Chlamydia trachomatis*, and ElaD by *E. coli* (Misaghi et al., 2006; Catic et al., 2007; Rytkönen and Holden, 2007; Rytkönen et al., 2007). Not surprisingly, recent studies revealed that *L. pneumophila* contains effector proteins with DUB activity that play an important role in remodeling the bacterial phagosome (Sheedlo et al., 2015).

### DUBs that cleave isopeptide bonds

In addition to the mART and PDE domains mentioned above, the SidE family proteins harbor a DUB domain located at its amino terminal end, characterized by the presence of the Cys_118_-His_64_-Asp_80_ catalytic triad found in many proteases (Sheedlo et al., 2015). The DUB activity of SidEs exhibits a preference for K63-linked poly-ubiquitin chains (Sheedlo et al., 2015). These DUBs are also active against Neddylation, indicating substrate promiscuity (Sheedlo et al., 2015). The DUB activity of SidEs plays a role in the enrichment of polyubiquitin to the LCV, as more vacuoles harboring the SidEs deletion mutant are positive in the association with polyubiquitinated species (Sheedlo et al., 2015). Yet, the DUB activity of SidEs is not required for maximal intracellular bacterial replication (Sheedlo et al., 2015); This DUB may function to release ubiquitin from modified proteins to provide a reaction precursor for the ligase activity conferred by the mART and PDE domains.

Another *L. pneumophila* effector protein LupA (Lpg1148) also harbors a DUB domain. LupA catalyzes the removal of ubiquitin from target proteins, a reaction that requires the predicted C-H-D catalytic triad (Urbanus et al., 2016). LupA rescues the yeast growth defect caused by the *L. pneumophila* effector protein LegC3; In addition, LupA removes ubiquitin modification from LegC3 when co-expressed in mammalian cells (Urbanus et al., 2016). Collectively, these observations suggest that the activity of LegC3 may depend upon on ubiquitination by one or more host E3 ligases, and LupA functions to inactivate it via specific deubiquitination (Urbanus et al., 2016). The biological role of LupA during bacterial infection requires further investigation.

### A DUB that cleaves the linkage induced by members of the SidE family effectors

**Figure 3:**
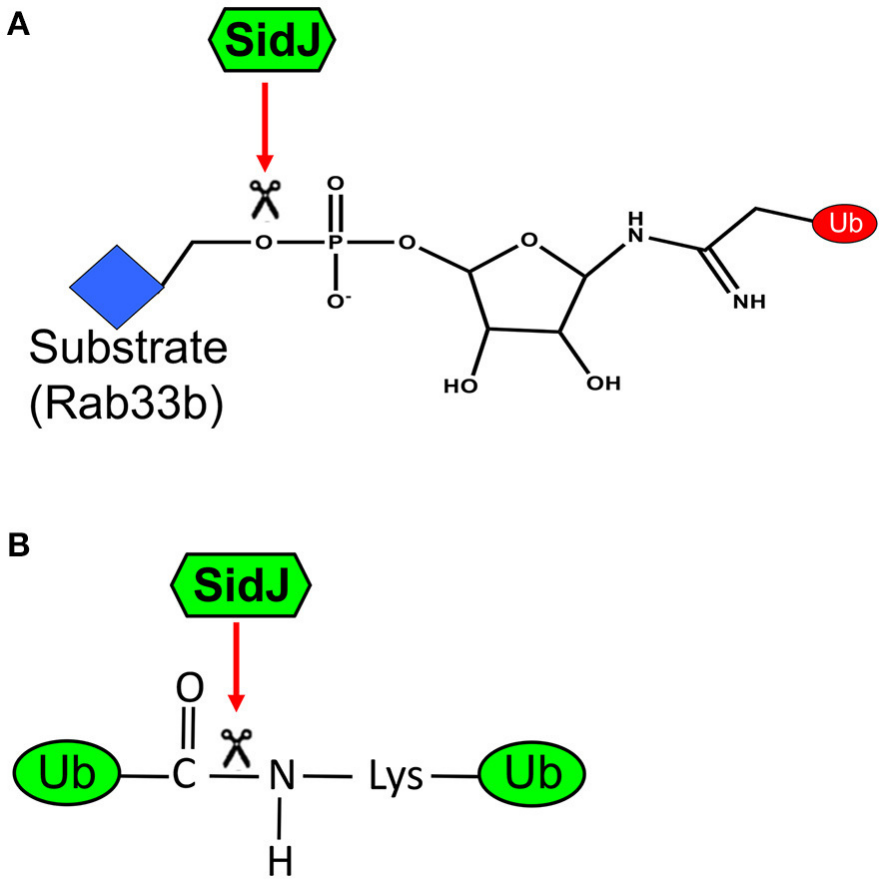
A diagram of the dual biochemical activities of SidJ. **(A)** Cleavage of phosphodiester bond by SidJ. SidJ specifically recognizes the phophosdiester bond that links phosphoribosylated ubiquitin to serine residue on the substrate. This activity allows the substrate to return to its unmodified form; it also produces phosphoribosylated ubiquitin, which may be further hydrolyzed by enzymes from either the host or the bacterium or both. **(B)** Cleavage of isopeptide bond by SidJ. SidJ recognizes and cleaves the isopeptide bond that links ubiquitin to lysine residue of the substrate or another ubiquitin moiety. This activity produces free ubiquitin. Since mutants that retain only one of these two activities have not been isolated, the importance of the classical DUB activity of SidJ is not clear.

One unique feature of *L. pneumophila* effectors is the regulation of one effector activity by another effector, with the latter being designated as metaeffector (Kubori et al., 2010). Such regulation is achieved by affecting protein stability exemplified by LubX and SidH and more commonly by effectors with opposite biochemical activities such as SidD and Lem3 that antagonize the activity of SidM and AnkX, respectively (Tan and Luo, 2011; Tan et al., 2011). Although, the biological significance is not known, regulation by direct protein-protein interactions between effectors has also been suggested (Urbanus et al., 2016). The activity of SidEs is regulated by another effector SidJ, which itself is also required for maximal bacterial intracellular growth (Liu and Luo, 2007). SidJ suppressed yeast toxicity of members of the SidE family (Havey and Roy, 2015; Jeong et al., 2015), suggesting that it may reverse the modification imposed by the ligases. Indeed, recombinant SidJ effectively removes ubiquitin from modified substrates such as Rab33b by cleaving the phosphodiester bond between ubiquitin and substrate protein (Qiu et al., 2017; Figure 3A). These results establish SidJ as a phosphodiesterase (PDE). Although, it has been reported that some DUBs cleave non-isopeptide bond like oxyester and thio-ester linkages, this is the first DUB known to cleave a phosphodiester linkage (Ronau and Hochstrasser, 2017). Substitution mutants failed to rescue SidEs-induced toxicity against eukaryotic cells also failed to complement the Δ*sidJ* mutant in infection. Without exception, these mutants have almost completely lost the DUB activity (Qiu et al., 2017). Thus, the DUB activity is responsible for the role of SidJ in *L. pneumophila* infection.

Surprisingly, SidJ also displays activity against isopeptide bond, and is able to deubiquitinate from proteins modified by the canonical pathway (Figure 3B). SidJ hydrolyzes K11, K33, K48, and K63-linked diubiquitin, but with a preference for the K63 linkage. Intriguingly, although it is sensitive to N-ethylmaleimide, a commonly used inhibitor that reacts with and blocks active thiol groups, mutations in any of the three cysteine residues did not detectably affect the activity of SidJ (Qiu et al., 2017), suggesting that SidJ is not a member of the thiol protease family. SidJ may represent a unique DUB which uses a novel catalytic mechanism to cleave both isopeptide and phosphoribosyl linkage. Whether these two activities are conferred by a single or two catalytic motifs are unknown. It is worth noting that SidJ purified from *L. pneumophila* but not from *E. coli* showed the DUB activity (Qiu et al., 2017), suggesting that SidJ needs co-factor(s) unique to *L. pneumophila* to function. The exact catalytic mechanism of SidJ and the nature of such co-factor(s) await further investigation.

Although, SidJ is constitutively expressed in broth-grown *L. pneumophila* (Liu and Luo, 2007), the quantity of protein translocated into host cells significantly increase as infection proceeds (Qiu et al., 2017; Figure 2B). The increasing ratios between SidJ and SidEs in infected cells render the DUB activity to become dominant at later infection phases, thus allowing temporal regulation of the activity of SidEs. Indeed, the amount of ubiquitinated Rab33b begins to decrease several hours after infection with wild type bacteria, but such decrease was delayed in infections using the Δ*sidJ* mutant (Qiu et al., 2017). Based on its ability to make SidEs undetectable from the LCV by immunostaining, it has been suggested that SidJ also spatially regulates the activity of SidEs (Jeong et al., 2015). Yet, the mechanism of such regulation, even if it exists, is unknown. The fact that the ligase activity of SdeA (likely other members of the SidE family, too) does not affect its cellular localization suggests that self-ubiquitination is not important for the association of SdeA with specific organelles. As a result, the DUB activity of SidJ unlikely plays a role in altering the cellular localization of SdeA.

Intriguingly, the amount of ubiquitinated substrates eventually decreases in cells infected with the Δ*sidJ* mutant, suggesting the existence of additional bacterial proteins or host enzymes capable of reversing ubiquitination induced by SidEs. Such enzymes from host cells may function with the putative endogenous ligases that catalyze NAD-dependent ubiquitination to regulate certain cellular processes.

## Concluding remarks

*L. pneumophila* encodes a large cohort of effectors to modulate the host ubiquitination system for its benefit, which emphasizes the importance of the ubiquitin network in the virulence of this pathogen. The diverse strategies ranging from functional mimicry of canonical E3 ligases or DUBs to mechanisms of completely different chemistry employed by this pathogen have deepened our understanding in not only bacterial pathogenesis but also in cell biology of the host cell. Despite the progress in biochemical characterization of these ubiquitin-editing effectors, our understanding of their role in the biogenesis of the LCV remains limited. A major challenge is that we know very little about the host proteins specifically targeted by these enzymes, let alone the biological significance of the modification imposed by these effectors. Given the complexity of the regulation of the ubiquitin network, it is anticipated that more Dot/Icm effectors involved in hijacking this signaling mechanism will be uncovered. A detailed understanding of their biochemical activities and the coordination of these effectors during bacterial infection will provide insights into both *L. pneumophila* pathogenesis and signaling in eukaryotic cells.

## Author contributions

JQ: prepared the first draft of the manuscript; Z-QL: initated the points to dicuss, revised and finalize the manuscript and prepared Figure 3.

### Conflict of interest statement

The authors declare that the research was conducted in the absence of any commercial or financial relationships that could be construed as a potential conflict of interest. The reviewer SP and handling Editor declared their shared affiliation.
